# A high-throughput qPCR system for simultaneous quantitative detection of dairy *Lactococcus lactis* and *Leuconostoc* bacteriophages

**DOI:** 10.1371/journal.pone.0174223

**Published:** 2017-03-24

**Authors:** Musemma K. Muhammed, Lukasz Krych, Dennis S. Nielsen, Finn K. Vogensen

**Affiliations:** Department of Food Science, University of Copenhagen, Frederiksberg C, Denmark; University of Torino, ITALY

## Abstract

Simultaneous quantitative detection of *Lactococcus* (*Lc*.) *lactis* and *Leuconostoc* species bacteriophages (phages) has not been reported in dairies using undefined mixed-strain DL-starters, probably due to the lack of applicable methods. We optimized a high-throughput qPCR system that allows simultaneous quantitative detection of *Lc*. *lactis* 936 (now *SK1virus*), P335, c2 (now *C2virus*) and *Leuconostoc* phage groups. Component assays are designed to have high efficiencies and nearly the same dynamic detection ranges, *i*.*e*., from ~1.1 x 10^5^ to ~1.1 x 10^1^ phage genomes per reaction, which corresponds to ~9 x 10^7^ to ~9 x 10^3^ phage particles mL^-1^ without any additional up-concentrating steps. The amplification efficiencies of the corresponding assays were 100.1±2.6, 98.7±2.3, 101.0±2.3 and 96.2±6.2. The qPCR system was tested on samples obtained from a dairy plant that employed traditional mother-bulk-cheese vat system. High levels of 936 and P335 phages were detected in the mother culture and the bulk starter, but also in the whey samples. Low levels of phages were detected in the cheese milk samples.

## Introduction

Strains of *Lactococcus* (*Lc*.) *lactis* are extensively used in the manufacture of most fermented dairy products, and *Leuconostoc* species are widely used as well. The associative growth of these two bacteria groups has been described as a synergistic functional relationship [1]. In the manufacture of cheese, *Lc*. *lactis* strains cause rapid acidification of milk through the production of lactic acid. The presence of *Lc*. *lactis* enhances the survival of *Leuconostoc* strains, which grow poorly in milk on their own [2,3]. In dairy fermentations, *Leuconostoc* strains were shown to influence multiple organoleptic properties of fresh and semi-hard cheese varieties (such as Tilsitter, Edam and Gouda) and unripened dairy products [2,4–6].

Industrial cheese fermentation vats are ideal environments for the multiplication of virulent bacteriophages (phages), since they are not inactivated by the heat treatment that is commonly used in the dairy industry [7–9]. Infection by phages of *Lc*. *lactis* results in partial or complete interruption of fermentation leading to significant economic losses due to longer processing time, waste of ingredients, reduced product quality and consistency, as well as growth of spoilage microorganisms and pathogens [10]. Infection by phages of *Leuconostoc* strains are generally less detrimental but might result in loss of the desired flavor and aroma of the final product, or lack of sufficient eye formation in, e.g., blue mold cheese.

Members of three *Lc*. *lactis* phage species [936 (now *SK1virus*), P335 and c2 (now *C2virus*)] are widely distributed in dairies across the world [11,12], many of which have been identified in relation to dairy fermentation problems [13]. All members of the 936 and c2 species investigated so far are virulent phages, while the P335 species consists of both virulent and temperate members [14,15]. Dairy *Leuconostoc* phages are primarily categorized into two main categories: group I infecting *Leuconostoc (Le*.*) mesenteroides* and II infecting *Le*. *pseudomesenteroides* [16]. With the exception of one temperate phage (φMH1) [17], all *Leuconostoc* phages identified so far are lytic phages.

Rapid, cost effective and sensitive phage detection methods are necessary to reduce phage outbreaks in the dairy industry. So far, the presence of phages in dairy manufacturing plants has been detected by a number of indirect and direct methods.

Indirect phage detection methods are based on detection of the effect caused as a result of infection of a bacterial strain by a phage. For instance, activity tests are based on assessment of a decrease in acid production [10]. Another indirect but rapid phage detection method, which is based on detection of changes in the electrical conductance of milk due to a decrease in lactic acid production when a phage infection occurs, has been shown to be applicable to dairy phages [18]. However, it requires a suitable bacterial host to grow on the microelectrode surface [10].

Direct phage detection methods are based on detection of the presence of phage particles or their components in a sample. Standard microbiological methods such as plaque assays and spot tests are relatively laborious, time consuming and have low throughput. They are often applied to milk or fermented products and cannot be used for undefined DL-starters, as they require pure indicator strains. PCR based methods do not depend on viral infectivity and could give a detection limit in the range of 10^3^–10^7^ phage particles mL^-1^ [19]. These methods have been successfully applied to various dairy samples [20–26]. The multiplex PCR assay designed by Labrie and Moineau to detect *Lc*. *lactis* 936, P335 and c2 phages in a single reaction allowed to increase throughput and reduce cost [21]. The universal PCR detection system designed by Ali *et al*. allowed qualitative detection of dairy *Leuconostoc* phages [16]. Increasing demands for quantitative, more sensitive, rapid and real-time monitoring of specific phages during the fermentation process has prompted the development of real-time quantitative PCR (qPCR) methods.

Real-time PCR assays for dairy phages were first reported in 2008 [27,28]. The Del Rio *et al*. study reported multiplex real-time PCR for successful quantitative detection and identification of *cos*- and *pac*-type *Streptococcus thermophilus* phages in milk samples [27]. The Merrall *et al*. study presented successful species-level classification of dairy *Lc*. *lactis* 936 and c2 phage groups [28]. Since then, several methods have been developed and used for detection and identification of dairy *Lc*. *lactis* 936, P335 and/or c2 phages and other lactic acid bacteria phages. These include not only successful quantification of 936 and c2 phage groups in aerosol and surface samples of a typical cheese manufacturing plant [29] but also a rapid detection of 936, P335 and c2 phage groups in dairy products in just about 2 hours [30], with detection limit reaching ~10^2^ phage particles mL^-1^. Real-time PCR detection of dairy *Leuconostoc* phages has so far not been reported.

Advances in genome sequencing and analysis have brought much more comprehensive view in the diversity of dairy phages and the databases keep increasing [31,32]. This highlights the need for an improvement of previously developed phage detection methods, preferably based on the state of the art high-throughput methods.

In this study, we introduced a high-throughput qPCR assay based on the BioMark HD System (Fluidigm, USA) for simultaneous quantitative detection of dairy *Lc*. *lactis* and *Leuconostoc* phages. The assay targets three most common *Lc*. *lactis* phages 936, P335 and c2, and *Leuconostoc* phages. We also included data obtained from analysis of phages in a dairy plant using the same DL-starter propagated in a traditional mother culture-bulk starter-cheese vat system.

## Materials and methods

### Primer design

Primers were designed using CLC Genomics Workbench primer tool (Qiagen, Aarhus) and synthesized at Integrated DNA Technologies (IDT, Germany). Databases were screened for conserved genes in the genomes of the phages. Sequenced genomes, available in public databases and obtained from in-house sources, were used to perform multiple sequence alignment. At least 90% primer-target identity was used to qualify primers. The primers’ specificity was tested *in silico* using primer-BLAST (NCBI, USA) with strict parameters (above four primer-unintended target mismatches within the last 10 bp at the 3' end, above six mismatches in total). The primers designed and optimized in this study are summarized in Table 1.

**Table 1:** Species-specific primers designed for phages attacking *L*. *lactis* and *Leuconostoc* species.

Assay	Model phage	Primer^1^	Sequence (5' = >3')	Target gene	Concentration (nM)	Ta^2^ (°C)
936	sk1	F	GCATTGTTCRGCTAAAACTTT	*terS*	250	55
		R	AGCTTCGTCATACGCCTTTAT		500	
P335	TP901-1	F	AAGCGTGGCATTGCATT	*dut*	250	55
		R	CAGGCTCTTTTGAGATGTTCA		250	
c2	P220	F	ACTAGCGGTGCATTTAATGAAC	*ter*	250	55
		R	GCGTCAGCCAAATCAATCTTATC		500	
*Leuconostoc*	LN04	F	TGGTATGGTTGCTTTDTATAAC	*pol*	500	55
		R	TAGTTTAAACTCRTCTTCCCA		500	

^*1*^F: forward, R: reverse

^*2*^Ta: annealing temperature

### PCR and qPCR assays

DNA from phages sk1, TP901-1, P220 and LN04 was used as standard for developing the assays for the respective 936, P335, c2, and *Leuconostoc* phages.

The primers annealing temperature was tuned with gradient PCR (SureCycler 8800, Agilent Technologies, USA) using 2x PCR Master Mix (Thermo Fisher Scientific, USA).

Real-time qPCR assays were performed using Fast SYBR Green Master Mix (Thermo Fisher Scientific) on 7500 Fast Real-Time PCR System (Applied Biosystems, USA). To test the optimum concentrations of the primers, identical reactions containing different concentrations of the forward and reverse primers, prepared essentially as described [33], were run using the following thermocycling conditions: initial stage at 50°C for 2 min, hot start at 95°C for 2 min; followed by 40 cycles of (i) 95°C for 15 sec, (ii) 55°C for 30 sec and (iii) 72°C for 30 sec. Serial tenfold dilutions of corresponding phage genomic DNA was used to generate standard curves. Subsequent to the amplification, a melting curve analysis was performed in order to distinguish putative nonspecific amplifications. Each reaction was prepared in duplicate and each assay was repeated at least three times.

PCR reactions for comparison of SYBR Green I (Thermo Fisher Scientific) and EvaGreen (Biotium, USA) detection chemistries were prepared as described above using the P220 phage DNA as template. Slopes and efficiencies were analyzed by setting the threshold level at 0.1. Each reaction was prepared in duplicate.

### High-throughput qPCR (HT-qPCR) assays

Preamplification (PreAmp) was performed using PreAmp Master Mix (Fluidigm) on a SureCycler 8800 (Agilent Technologies) according to the manufacturer instructions (Fluidigm PN 68000088 K1, User Guide). A standard mix (pooled standard mix or PSM) was prepared by pooling DNA from sk1, TP901-1, P220 and LN04 in equimolar concentrations. The PreAmp thermocycling conditions were as follows: hot start at 95°C for 5 min, 14 cycles of (i) 95°C for 15 sec, (ii) 55°C for 30 sec and (iii) 60°C for 1 min, followed by a holding stage (60°C for 10 min).

The HT-qPCR assay was performed on the BioMark HD System (Fluidigm) according to the suppliers’ protocol (Fluidigm PN 100–7717 B1) using the Flex Six IFC (Fluidigm) chip and the following thermal conditions: thermal mix (1 cycle at 25°C for 360 sec, 1 cycle at 70°C for 360 sec), hot start (1 cycle 95°C for 300 sec), cycling (40 cycles of (i) 95°C for 15 sec, (ii) 55°C for 30 sec, and (iii) 60°C for 30 sec). Melting curve was applied using default settings.

### Dairy samples screening

Samples of milk, mother culture, bulk starter, and whey from the first and the last productions, collected in sterile containers, were delivered frozen to University of Copenhagen. After the first thawing, samples were divided in aliquots of 2 x 10 mL and stored at -60°C prior to analysis.

Samples were thawed in water bath (~30°C) and NaCl was added to a final concentration of 1 M. The NaCl-sample mixture was incubated at 4°C for 1 h and the pH adjusted to 4.0–4.6. The mixture was centrifuged for 15 min at 15,000 x g. Phage particles were concentrated by polyethylene glycol-precipitation (10% final concentration) for 1 h at 4°C and pelleted by centrifugation for 15 min (12,500 x g) at 15°C. The phage pellet was treated with DNase I overnight at 37°C (50 units mL^-1^ final concentration) (Sigma Aldrich, USA). Viral capsids were digested with Proteinase K (20 μg mL^-1^ final concentration) (Sigma Aldrich) at 55°C for one hour, in the presence of EDTA (Sigma Aldrich) and SDS (Sigma Aldrich) (final concentrations of 10 mM and 1%, respectively). Extraction of viral DNA was performed using the GenElute Bacterial Genomic DNA Kit (Sigma Aldrich) as described by the manufacturer. Viral DNA was eluted with a modified double elution procedure (2 x 100 μL elution buffer).

Sample DNA extracts were diluted 100-fold prior to PreAmp, which was performed as described above. Subsequent to PreAmp, samples were analyzed on Flex Six IFC as technical triplicates and biological duplicates.

### Data analysis

The overall performance and predictive power of the qPCR assays were evaluated from the equation of the linear regression lines, along with the squared correlation coefficients (R^2^). Whenever needed, amplification efficiencies were calculated from the slopes using the equation E = 10^−1/slope^ [34]. The amount of phage genomes in the samples was calculated from the Ct values corresponding to standard calibration curves. Data is expressed as the mean values and standard deviations from a total of six measurements (2 biological x 3 technical replicates).

## Results and discussion

### 1. Primer designing and PCR conditions

The main objective of this study was to develop a system for simultaneous quantification of 936, P335, c2, and *Leuconostoc* phage species. Phages sk1, TP901-1, P220 and LN04, respectively, were used as model phages for developing and testing the assays. Using genomes available in the databases and from own collection we found four candidate genes presenting highly conserved sequences: *terS* encoding putative small terminase subunit of sk1, *dut* encoding the putative dUTPase of TP901-1, *ter* encoding the putative terminase of P220, and *pol* encoding the putative DNA polymerase of LN04. All primers have been designed based on these conserved genetic features.

It was confirmed by pairwise comparison that there was a minimum of 90% identity between primers and virtually all analogous sequences on the target gene accessible at the moment (S1 File). A total of 74, 18, 12 and 15 full genome sequences of the 936, P335, c2 and *Leuconostoc* group of phages were used, of which 54, 16, 2 and 8, respectively, were available in GenBank, while the rest were in-house unpublished genome sequences. It is therefore reasonable to assume that the high identity between the primers and target sequences may increase the chance for the primers to target more, yet to be identified phages genomes belonging to the same group.

The effect of different primer concentrations on the kinetics of amplification was tested as essentially described [33]. The amplification results were compared with respect to the kinetics of amplification and dissociation. The objective was to achieve relatively early amplification signal (Ct) coupled with a clear single peak on the dissociation curve [35]. The results showed that certain primer formulations, summarized in Table 1, yielded early amplification signals with high-specificity of binding, characterized by no apparent evidence of primer cross/self hybridization and/or secondary binding. This proved the utility of the primers for the intended qPCR assays.

### 2. Evaluation of amplification efficiency

The optimized primer concentrations were then used to amplify serially diluted DNA of the target phages with qPCR. Assessment of amplification plots and standard curves (constructed from Ct values plotted against starting quantity of DNA) indicated linearity over at least 5 logs in all the tests. Using a constant Ct threshold of 0.1, all the assays (except a single P335 assay) showed standard curve slopes within an acceptable range of -3.6 to -3.1, corresponding to amplification efficiencies of 90% to 110% (Table 2). The exceptional P335 assay showed slope of -3.65 (~88% amplification efficiency).

**Table 2:** Performance of individual qPCR assays.

	sk1	TP901-1	P220	LN04
Slope	-3.36±0.1	-3.4±0.2	-3.40±0.1	-3.37±0.1
Y-intercept	34.3±1.2	44.2±9.6	36.4±1	34.8±1.9
R^2^	1.00±0.01	0.99±0.01	0.99±0.01	0.99±0.00
Efficiency (%)	98.4±4.5	97.3±7.9	96.8±2.7	98.0±5.7

Detection with SYBR Green I chemistry

Performance parameters calculated based on at least 3 independent experiments

The squared correlation coefficients (R^2^) for the standard curves were within the range of 0.99 and 1.0 (Table 2), demonstrating robust predictive power of the assays to quantify phages. The theoretical detection limits were calculated to be one phage DNA molecule in about 34 to 44 cycles depending on the assay (Table 2). Variability between the technical replicates increased when the number of genome copies dropped below 100. This is in agreement with the detection limit obtained in other studies based on various platforms and chemistries [27,30].

**Fig 1:**
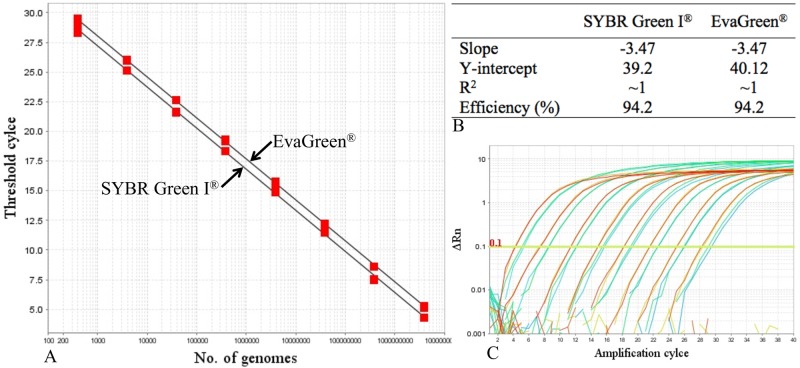
Performance of SYBR Green I and EvaGreen detection chemistries during qPCR assays. (A) Standard curves generated from amplification of serially diluted *L*. *lactis* phage P220 genome detected with the corresponding chemistries; (B) the corresponding performance parameters; and (C) amplification plots detected with SYBR Green I (brown) and EvaGreen (green).

Despite being very common dye-based detection chemistry, SYBR Green I is not compatible with some High Throughput qPCR systems, such as the Fluidigm Dynamic Array system. The latter performs optimal with EvaGreen, which has been found to be stable under PCR reactions [36]. We therefore tested both detection chemistries on the overall performance of the assays. The results indicated that both chemistries detected the amplification with equally high efficiencies (94.2% in both cases), except that EvaGreen yielded slightly delayed signal than the other (Fig 1). Linearity between Ct values and starting quantity of DNA was equally high in both cases and encompassed as many as 8 logs (Fig 1). This finding suggests that both dyes produce only minimal effect on the overall assay performance and highlights the possibility of making the data generated by both chemistries comparable.

### 3. Optimization of the HT-qPCR system

The main challenge in designing the assay using HT-qPCR system was to adjust similar dynamic ranges of detection. An important consideration was that the maximum copies of each genome prior to PreAmp should correspond to the highest possible quantification limit on the Flex Six IFC reaction chamber. Preliminary experiments showed that PSM containing ~1 x 10^6^ template copies μL^-1^ exceed the quantification threshold due to 14 cycles of PreAmp. In such cases, the easiest solution would be either to reduce the number of PreAmp cycles, or further dilute the PreAmp. However, in environments presenting large disproportion in the relative abundance of different phages, assays optimized based on fewer PreAmp cycles could compromise sensitivity. This is true for the dairy environment, where some phages (such as 936) are often highly abundant, while others (such as c2) are less represented. On the contrary, too many PreAmp cycles could favor less represented phages, since abundant phages may exceed the highest quantification limit. Our results indicate that 14 PreAmp cycles, which facilitates preamplification of both abundant and less represented phages to the intended dynamic range is the optimal setup for detection of selected phages in dairy samples.

Further investigations showed that PSM containing nearly 9 x 10^4^ template copies μL^-1^ corresponds to the highest quantification limit on the IFC RC. This is equivalent to ~1.1 x 10^5^ template copies per genome in the PreAmp reaction. Standard curve generated from log dilutions of this PSM subjected to 14 cycles of PreAmp demonstrated the existence of linear relationship between Ct values and starting amount of template across five log dilutions to the minimum (*i*.*e*., ~1.1 x 10^5^ to ~1.1 x 10^1^ template copies) (Table 3, S1 File).

**Table 3:** Performance of qPCR assays as components of the high-throughput system.

	sk1	TP901-1	P220	LN04
Slope	-3.3±0.1	-3.3±0.1	-3.3±0.2	-3.4±0.2
Y-intercept	22.4±0.6	22.6±0.6	22.4±0.6	24.2±1.0
R^2^	0.99±0.01	0.99±0.01	0.99±0.01	0.99±0.00
Efficiency (%)	100.4±2.8	99.4±2.6	102.5±7.3	96.3±7.8

Detection with EvaGreen chemistry

Performance parameters calculated based on four independent experiments

The 936 and c2 assays often showed linearity across six logs, which is consistent with higher linearity of the same two assays observed based on SYBR Green I chemistry. The PCR efficiencies ranged from 96.15±6.2% to 100.1±2.56 (Table 3) with Pearson's correlation coefficients (R^2^) of 0.99±0.00 (for 936 and c2) or 0.99±0.01 (for P335 and *Leuconostoc*).

Notably, the dynamic ranges of detection of all the assays were comparable covering at least 5-logs (using 14 PreAmp cycles). The method detection limit yielded ~11 copies per PreAmp reaction. The theoretical dynamic range of detection is, therefore, between ~9 x 10^7^ to ~9 x 10^3^ phage particles mL^-1^ without considering sample concentration steps. Any additional concentration step is expected to cause proportional reduction in the limit of detection. In general, this qPCR system provides the best combination of primers, efficiency and dynamic range of detection for simultaneous quantitative detection of the four groups of phages reported to date.

### 4. Advantages and limitations

The qPCR system allows for simultaneous quantitative detection of the genomes of the four phage groups in the given dynamic detection range. Component assays are both flexible and efficient (not limited to a single platform or detection chemistry and can be run individually and simultaneously). Achievement of comparable dynamic detection range for all the assays likely improves reproducibility and data analysis. The system is without a doubt highly applicable for research use, although its implementation for routine monitoring of dairies can be challenging due to the need for costly equipment and trained personnel.

However, in collaboration with researchers it is possible to apply it for large-scale screening of the dairy environment prior to, or during some critical process (for instance factory design or process changes, etc.). It should be noted that individual assays have wider dynamic detection range compared to the HT-qPCR system, which is probably inherent to PCR systems with preamplification step.

The assays enable detection of nearly the entire genome sequences of the corresponding phages available in GenBank along with several sequences from in-house sources. Through *in silico* analysis, we confirmed that the respective primer pairs show a minimum of 90% complementarity to 112 936 genomes (total = 114), 16 P335 genomes (total = 18), 12 c2 genomes (total = 12) and 12 *Leuconostoc* phages genomes (total = 15) (S1 File). The genomes of the 936 phages not covered by the primers include SCS and SDN, which are in-house unpublished genomes, and of P335 phages include 4268 and phi31, which are known to be devoid of the *dut* gene. Of *Leuconostoc* phages, phiLN23 was the only phage, for which the primers show relatively low complementarity [68% (the forward) and 57% (the reverse)]. For phages LN-8, LN-9 and LN25, the primers exhibit a minimum of 86% complementarity but some of the mismatches are located towards the 3’ end of the forward primer, in particular.

A general limitation of qPCR for quantification of dairy phages is that it cannot distinguish whether DNA comes from active phages or prophages. Bacterial DNA in dairy samples likely contains prophages due to the wide spread nature of lysogeny in *Lc*. *lactis* strains [37]. This is particularly relevant when dealing with the P335 quasi-species, which consist of both temperate and virulent members [8,14,38,39]. For instance, Kleppen *et al*. illustrated that high quantity of P335 DNA could be detected in bulk starter samples when no DNase treatment was performed, otherwise no P335 phage DNA would be detected [40]. In the former case, the DNA originates from lysogenic starter strains lysed by virulent phages [21,40]. Furthermore, unpacked DNA of virulent phages can also be present in dairy samples [23]. Thus, accurate quantification of problematic phages requires efficient removal of intact bacterial cells from the samples and incorporation of effective DNase treatment step prior to extraction of metavirome DNA.

### 5. Species determination and quantification of phages in a dairy

**Fig 2:**
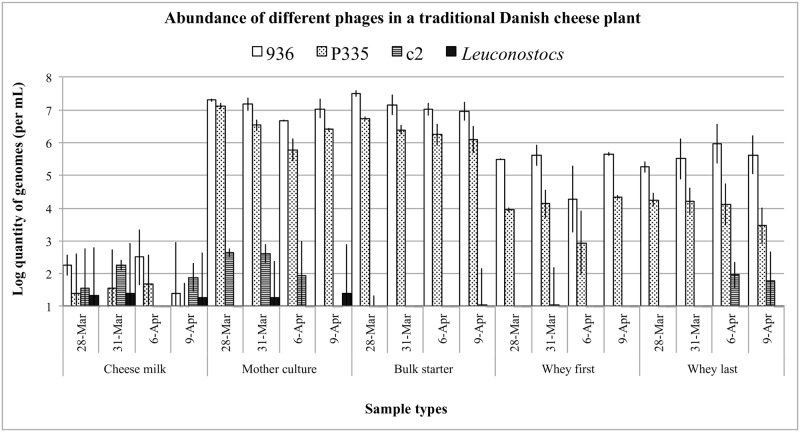
Quantities of *L*. *lactis* 936, P335, c2 and *Leuconostoc* groups of phages in a dairy plant. The quantity of phage genomes detected in one mL of cheese milk, mother culture, bulk starter and first and last wheys on five representative production days (on March and April, 2014) is shown. This was calculated from (determined as) the amount of genomes detected in 1.25 μl of 100-fold diluted phage DNA extract using HT-qPCR on the BioMark HD system.

The qPCR system was tested on milk, mother culture, bulk starter, and whey samples obtained from a dairy using DL-starters. 936 and P335 phages were detected in high numbers in almost all the tested samples, however, with significantly smaller quantity in milk samples (Fig 2). Very few c2 and *Leuconostoc* phages were detected in some milk, mother culture, and last whey (only c2) samples, indicating that these phages were less prevalent in the dairy.

In general, 936 phages appeared to be more frequent and abundant than the other phages in the dairy environment. The quantity of 936 genomes varied between 10^6^ and 10^8^ mL^-1^ of mother culture and bulk starter, and between 10^5^ and 10^6^ mL^-1^ of first and last wheys. This is not surprising since the 936 species is known for long time to be the most frequently isolated phage in dairy plants in different areas of the world [40–46].

The heterogeneous P335 quasi-species was also abundant in the dairy. The quantity of P335 genomes varied between 10^6^ and 10^7^ mL^-1^ of mother culture and bulk starter, and between 10^4^ and 10^5^ mL^-1^ of first and last wheys. Since samples were subjected to DNase I treatment, the majority of the P335 phages detected were likely to be virulent or induced phages.

Since the P335 primer set does not target the 4268 phage (as it does not contain the dUTPase gene) [11], it seems reasonable to assume that the actual P335 phage content of the samples could be higher than analyzed.

The quantity of c2 phages appeared to be mostly low in the dairy, which is in agreement with most lactococcal phage isolation studies [40,42–46] but also contrasts with few [47,48]. The quantity of *Leuconostoc* phages was also low. In mixed-strain DL-starters, strains of *Lc*. *lactis* represent the majority, whereas strains of *Leuconostoc* species constituted to 1–10% of the total bacteria count [4,6]. The low quantity of *Leuconostoc* phages in the present dairy could be due to the fact that the proportion of *Leuconostoc* strains in the starter cultures was low or that starter cultures were simply not infected.

Overall, data suggest that phages belonging to the 936 and P335 group are more frequent in the present dairy than c2 and *Leuconostoc* phages. Only few phages are present in raw milk, which suggest that milk is not the principal source of phage contamination to the dairy environment. The 936 and P335 phages are present at high levels already in the mother culture stage, which contaminates the bulk starter and, subsequently, the cheese vats and the remaining environment.

## Conclusion

To our knowledge, this is the first HT-qPCR system for simultaneous quantitative detection of dairy *Lc*. *lactis* and *Leuconostoc* species phages. The need for designing and optimizing new primers was (i) to accommodate for the exponentially grown number of genome sequences, (ii) to select for primers with comparable size and thermal properties, and (iii) to develop assays with comparable efficiencies and dynamic ranges of detection. The HT-qPCR system provides the best combination of primers, efficiency and dynamic range of detection for simultaneous quantitative detection of 936, P335, c2 and *Leuconostoc* species phages.

## Supporting information

S1 File Data underlying the findings in this study. Additional details on the development, optimization and validation of the qPCR assays discussed in the paper are provided in eight different sections, including Figures and Tables.
(PDF)
