# Supplementary material for: A high-throughput qPCR system for simultaneous quantitative detection of dairy Lactococcus lactis and Leuconostoc bacteriophages
Source: PLoS One. 2017 Mar 24;12(3):e0174223. doi: 10.1371/journal.pone.0174223 (PMC5365131; doi:10.1371/journal.pone.0174223)
Supplement: S1 File — Additional details on the development, optimization and validation of the qPCR assays discussed in the paper are provided in eight different sections, including Figures and Tables. (PDF) [file pone.0174223.s001.pdf]

Information Underlying the Manuscript 'A High-throughput qPCR System for Simultaneous Quantitative Detection of Dairy *Lactococcus lactis* and *Leuconostoc* Bacteriophages'

1. Experimental design

Definition of control and experimental groups

Standards: serial ten-fold dilution of known phage DNA used to establish calibration curves. Pooled standard mix (PSM) is a mixture of DNA from sk1 (936 type phage), TP901-1 (P335 type phage), P220 (c2 type phage) and phiLN04 (*Leuconostoc* phage).

Non-template controls (NTCs): samples that contain all the components of a PCR reaction except intentionally-added nucleic acid and, instead, contain an equal volume of sterile milliQ water.

Number within each group

Serial five ten-fold dilutions of the PSM constituted the standards. This was carried out for a total of three times (three independent experiments). Each independent experiment also comprised of at least one NTC. All the experiments were carried out by the investigator's laboratory.

Authors' contributions

Conceptualization: Musemma K. Muhammed, Lukasz, Krych, Dennis S. Nielsen, Finn K. Vogensen
Methodology: Musemma K. Muhammed, Lukasz Krych, Dennis S. Nielsen, Finn K. Vogensen
Resources: Musemma K. Muhammed, Lukasz Krych, Dennis S. Nielsen, Finn K. Vogensen
Formal analysis: Musemma K. Muhammed, Lukasz Krych
Investigation: Musemma K. Muhammed, Lukasz Krych
Data curation: Musemma K. Muhammed, Lukasz Krych, Dennis S. Nielsen, Finn K. Vogensen
Validation: Musemma K. Muhammed, Lukasz Krych, Dennis S. Nielsen, Finn K. Vogensen

Visualization: Musemma K. Muhammed, Lukasz Krych
Draft manuscript preparation: Musemma K. Muhammed
Manuscript review and editing: Musemma K. Muhammed, Lukasz Krych, Dennis S. Nielsen, Finn K. Vogensen
Funding acquisition: Finn K. Vogensen, Dennis S. Nielsen
Project administration: Finn K. Vogensen
Supervision: Finn K. Vogensen, Dennis S. Nielsen

2. Sample description, volume and processing procedures

Cheese milk. Milk intended for cheese production

Mother culture. A bacterial starter culture used for preparation of bulk starter through inoculation of 1% to the heat-treated milk.

Bulk starter. A bacterial starter culture made from mother culture and intended for inoculation into cheese milk to a final concentration of 1%.

First whey. Whey sample sampled from the first production of a sampling day immediately before drainage of whey.

Last whey. Whey sample sampled from the last production of a sampling day immediately before drainage of whey.

The samples, collected in sterile containers, were delivered frozen to University of Copenhagen. After thawing, samples were divided in aliquots of 2 x 10 mL and stored at -60°C prior to analysis. Samples were thawed in water bath (~30°C) and 1 M NaCl added. The NaCl-sample mixture was incubated at 4°C for 1 h and the pH adjusted to 4.0–4.6. The mixture was centrifuged for 15 min at 15,000 x g. Phage particles were concentrated by 10% polyethylene glycol 6000 (PEG 6000) (Sigma Aldrich, USA) for 1 h at 4°C and pelleted by centrifugation for 15 min (12,500 x g) at 15°C. The phage pellet was treated with DNase I for overnight at 37°C (50 units mL^{-1} final concentration) (Sigma Aldrich, USA). Viral capsids were digested with 20 µg mL^{-1} Proteinase K (Sigma Aldrich) at 55°C for one hour, in the presence of EDTA (Sigma

Aldrich) and SDS (Sigma Aldrich) (final concentrations of 10 mM and 1%, respectively). Viral DNA was finally extracted (see below).

3. Nucleic acid extraction

Extraction of viral DNA was performed using the GenElute Bacterial Genomic DNA Kit (Sigma Aldrich) as described by the manufacturer. Elution was carried out by a modified double elution procedure (2 x 100 µL elution buffer). The concentration of the extracted DNA was, whenever needed, measured with Qubit 2.0 fluorometer and Qubit HS kit (Invitrogen, USA), which detects target-specific fluorescence using state-of-the-art dyes selective for dsDNA.

5. qPCR target information

S1 Table. qPCR target information with sequence accession and amlicon length

**Assay**	**Target gene**	**Sequence accession No**	**Amplicon length (bp)**
936	sk1p01 (*terS*)	NP_044947.1	65
P335	*dut*	NP_044947.1	100
c2	l12 (c2)/ bIL67_gp06 (bIL67)	NP_043560.1 (c2)/ NP_042313.1 (bIl67)	102
Leuconostoc species	phiLN04_07 (*pol*)	NP_044947.1	157

***In silico* specificity screen**

The specificity of primers to the targets was tested *in silico* using primer-BLAST (NCBI, USA) with strict parameters (above four primer-unintended target mismatches within the last 10 bp at the 3' end, above six mismatches in total). With these parameters, unintended targets were not detected.

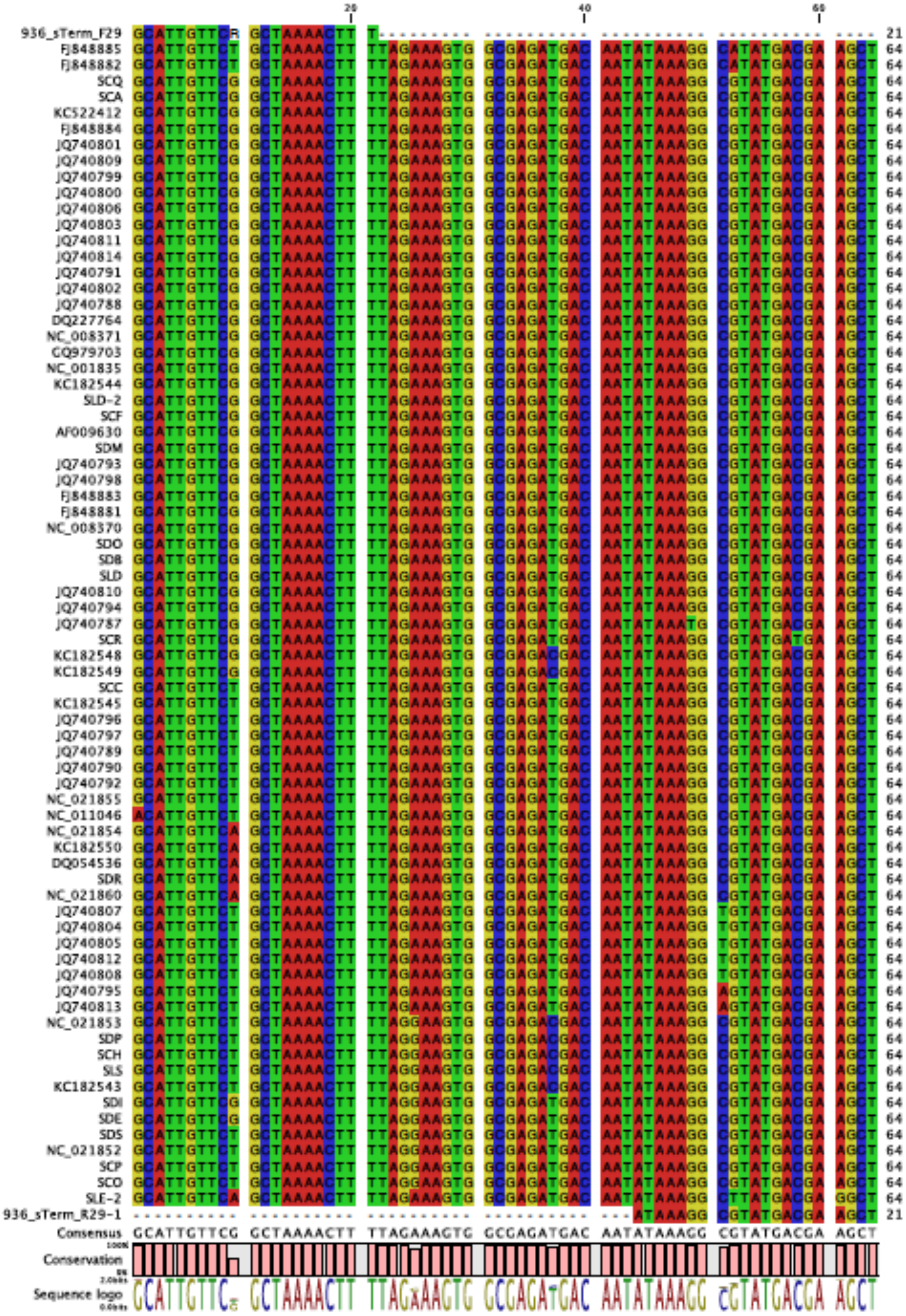

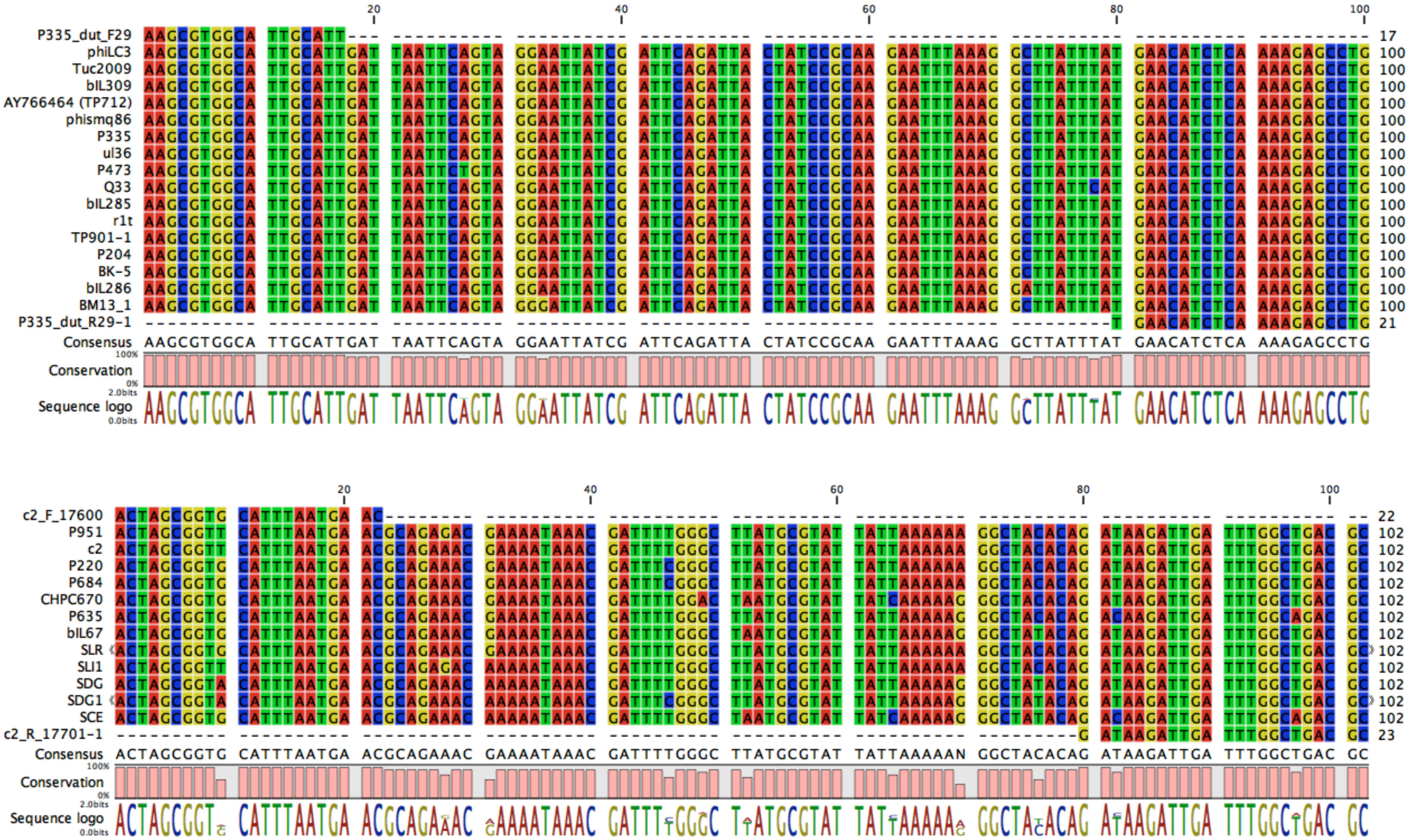
20
40
60
80
100
P335_dut_F29
phiLC3
Tuc2009
bIL309
AY766464 (TP712)
phismq86
P335
ul36
P473
Q33
bIL285
r1t
TP901-1
P204
BK-5
bIL286
BM13_1
P335_dut_R29-1
17
100
21
Consensus
AAGCGTGGCA TTGCATTGAT TAATTCAGTA GGAATTATCG ATTCAGATTA CTATCCGCAA GAATTTAAAG GCTTATTTAT GAACATCTCA AAAGAGCCTG
Conservation
100%
0%
Sequence logo
2.0bits
0.0bits
c2_F_17600
P951
c2
P220
P684
CHPC670
P635
bIL67
SLR
SLI1
SDG
SDG1
SCE
c2_R_17701-1
22
102
23
Consensus
ACTAGCGGTG CATTTAATGA ACGCAGAAAC GAAAATAAAC GATTTTGGGC TTATGCGTAT TATTAAAAAN GGCTACACAG ATAAGATTGA TTTGGCTGAC GC

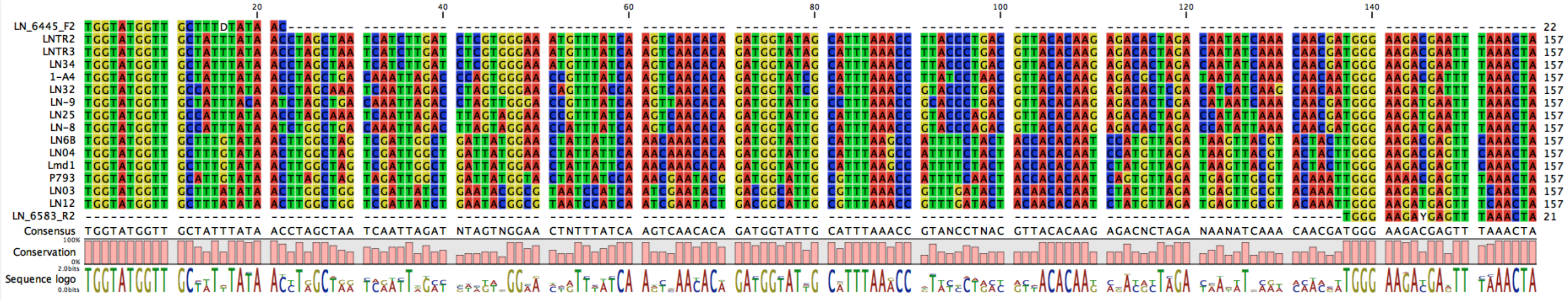

S1 Fig. Alignment of primers and target 936, P335, c2 and *Leuconostoc* phage genomes, respectively. The first and the last sequences in the respective alignments represent the forward and reverse primers, respectively, while the remaining sequences represent the target regions in the genomes of the phages. Alignments were run on CLC Genomics Workbench 7.0.4 (Qiagen, Denmark) 'Create Alignment' tool using default parameters.

Forward primer		1	Reverse primer		1
	1			1	
phiLC3	2	100.00	phiLC3	2	100.00
Tuc2009	3	100.00	Tuc2009	3	100.00
bIL309	4	100.00	bIL309	4	100.00
TP712	5	100.00	TP712	5	100.00
phismq86	6	100.00	phismq86	6	100.00
P335	7	100.00	P335	7	100.00
ul36	8	100.00	ul36	8	100.00
P473	9	100.00	P473	9	100.00
Q33	10	100.00	Q33	10	100.00
bIL285	11	100.00	bIL285	11	100.00
r1t	12	100.00	r1t	12	100.00
TP901-1	13	100.00	TP901-1	13	100.00
P204	14	100.00	P204	14	100.00
BK-5	15	100.00	BK-5	15	100.00
bIL286	16	100.00	bIL286	16	100.00
BM13_1	17	100.00	BM13_1	17	100.00

P335

Forward primer		1	Reverse primer		1
	1			1	
P951	2	95.45	P951	2	100.00
c2	3	95.45	c2	3	100.00
P220	4	100.00	P220	4	100.00
P684	5	100.00	P684	5	100.00
CHPC670	6	100.00	CHPC670	6	100.00
P635	7	100.00	P635	7	91.30
bIL67	8	100.00	bIL67	8	100.00
SLR	9	100.00	SLR	9	100.00
SLI1	10	95.45	SLI1	10	100.00
SDG	11	95.45	SDG	11	100.00
SDG1	12	95.45	SDG1	12	100.00
SCE	13	100.00	SCE	13	91.30

c2

Forward primer		1	Reverse primer		1
	1			1	
LNTR2	2	95.45	LNTR2	2	95.24
LNTR3	3	95.45	LNTR3	3	95.24
LN34	4	95.45	LN34	4	95.24
LN32	5	90.91	LN32	5	95.24
1-A4	6	95.45	1-A4	6	95.24
LN25	7	90.91	LN25	7	95.24
LN-8	8	86.36	LN-8	8	95.24
LN-9	9	86.36	LN-9	9	95.24
LN6B	10	100.00	LN6B	10	95.24
LN04	11	100.00	LN04	11	95.24
Lmd1	12	100.00	Lmd1	12	95.24
P793	13	95.45	P793	13	95.24
LN03	14	100.00	LN03	14	95.24
LN12	15	100.00	LN12	15	95.24
LN23	16	68.18	LN23	16	57.14

Leuconostoc

S2 Fig. Pairwise comparison between primers and target P335, c2 and *Leuconostoc* phage genomes. Only the region that is supposed to from a complex with the primers during the annealing stage of the PCR reaction was selected and used for the *in silico* comparison.

6. qPCR oligonucleotides

Primers were designed and ordered at Integrated DNA Technologies (IDT, Germany). Primers were purified with standard desalting purification method.

S2 Table. Primer sequences

Assay	Primer	Name	Sequence (5'=>3')
936	F	936_sTerm_F16	GCATTGTTCRGCTAAAACTTT
	R	936_sTerm_R16	AGCTTCGTCATACGCCTTTAT
P335	F	P335_dut_F29	AAGCGTGGCATTGCATT
	R	P335_dut_R29	CAGGCTCTTTTGAGATGTTCA
c2	F	c2_F_17600	ACTAGCGGTGCATTTAATGAAC
	R	c2_R_17701	GCGTCAGCCAAATCAATCTTATC
Leuconostoc spp	F	LN_6445_F2	TGGTATGGTTGCTTTDTATAAC
	R	LN_6583_R2	TAGTTTAAACTCRTCTTCCCA

7. qPCR protocol

Complete reaction conditions

Reaction volume and amount of DNA. For preamplification (PreAmp) of target DNA, a total of 5 µl of PreAmp reaction, consisting of 3.75 µL of PreAmp master mix (Fluidigm, Product # 100-5580) plus 1.25 µL of pooled standard DNA mix (PSM), was prepared. For the qPCR analysis, a total of 4 µl reaction containing 1.8 µl of pre-amplified and exonuclease I treated DNA was prepared. Of this, 3 µl was loaded per FLEXsix IFC (Product # 100-7485) inlet. Additional details can be found in Fluidigm PN 68000088 K1 User Guide.

S3 Table. Primer concentrations employed

**Assay**	**Primer concentrations (µM)**
936	2.5/2.5
P335	2.5/2.5
c2	2.5/2.5
Leuconostoc species	2.5/2.5

Polymerase identity and concentration. Sso7d-fusion polymerase was used, which is supplied as part of the SsoFast™ EvaGreen® Supermix with low ROX™ (2X). http://www.bio-rad.com/webroot/web/pdf/lsr/literature/10014647A.pdf. To our knowledge, the actual concentration of the polymerase has not been made public, nor the concentrations of the othe PCR components (Mg2+, dNTPs, etc.).

Manufacturer of plates/tubes and catalog numbers

PCR reactions for PreAmp of the PCR targets were prepared on Thermo-Fast® 96, Non-Skirted plates (ABgene, UK, Cat# AB-0600). The high-throughput qPCR assays were carried out using the FLEXsix Gene Expression IFC (Fluidigm, Product # 100-7485).

Complete thermocycling parameters

PreAmp conditions. 95°C for 5 min, 14 cycles of (i) 95°C for15 sec, (ii) 55°C for 30 sec and (iii) 60°C for 60 sec, and final elongation at 60°C for 10 min.

qPCR assay conditions. thermal mix (1 cycle at 25°C for 360 sec, 1 cycle at 70°C for 360 sec), hot start (1 cycle 95°C for 300 sec), cycling (40 cycles of (i) 95°C for 15 sec, (ii) 55°C for 30 sec, and (iii) 60°C for 30 sec) (on the BioMark HD System (Fluidigm, USA)).

Manufacturer of qPCR instrument

Fluidigm Corporation (CA, USA)

8. qPCR validation

Specificity was assessed by *in silico* specificity screen and sequence alignment (as described earlier), gradient PCR and melting curve analysis (see below).

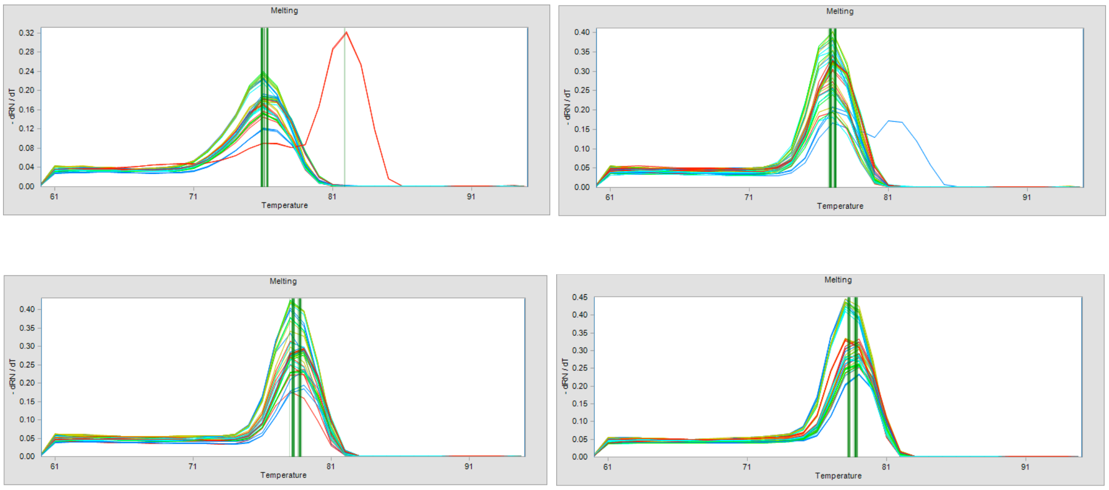

S3 Fig. Melting curves of the corresponding 936 (upper left), P335 (upper right), c2 (lower left) and *Leuconostoc* phage (lower right) amplicons.

Ct of the NTCs

S4 Table. Average Ct values and standard deviations of NTCs of positive amplification plots*

Assay	Mean Ct±SD
936	24.0±1.97
P335	24.8±3.06
c2	24.8±4.78
Leuconostoc species	23.9±0.75

**Calculation of mean Ct for NTCs did not take into account NTCs that did not give amplification plots within the given number of cycles. These NTCs account for more than half of the total number of NTCs analyzed*

Low Ct values of the NTCs are related to incorporation of 14 cylces of PreAmp prior to the actual qPCR assays. Assuming ~100% PCR efficiency, 14 cycles of PreAmp amplifies each target DNA added to the reaction by more than 16000 fold. This eventually leads to appearance of amplification plots many Ct cycles (which can be quantified, if need be) earlier than they do in conventional qPCR assays.

Calibration curves

Calibration curves were analyzed without considering the increase in the amount of DNA due to 14 cycles of PreAmp. Meaning that only the initial amount of DNA copies added to the PCR reactions were used to analyze the calibration curves. In our experience, this doesn't influence the slope of the calibration curve and hence the efficiency. However, it influences the y intercept. Thus, it should be considered that 14 cycles of PreAmp would increase the number of DNA copies by >16000 fold (i.e., initial number of DNA copies x 2^{14}). If we considered this increase in the number of DNA copies in the analysis, this would shift the y intercept by at least five points.

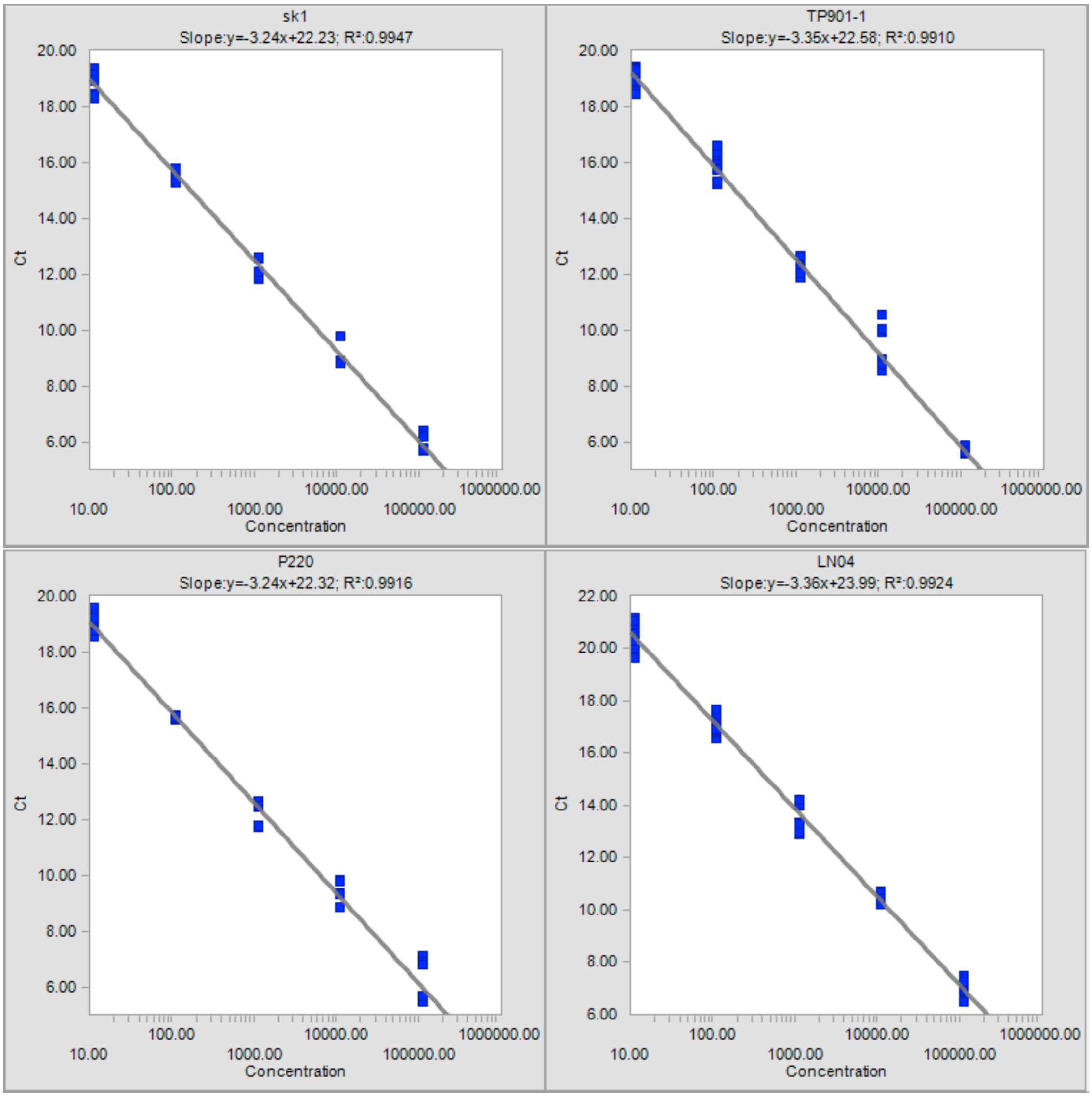

S4 Fig. Calibration curves of the qPCR assays. This is when data from the three independent experiments were combined and analyzed using the Fluidigm Real-Time PCR Analysis Software v 4.1.3.

S5 Table. Calibration curve slopes, y intercepts and efficiencies. This is when data from the three independent experiments were analyzed separately using the Fluidigm Real-Time PCR Analysis Software v 4.1.3. The mean slopes and standard deviations were subsequently calculated.

**Assay**	**Slope (±SD)**	**Y intercept (±SD)**	**Efficiency % (±SD)**
**936**	-3.3±0.1	22.4±0.6	100.4±2.8
**P335**	-3.3±0.1	22.6±0.6	99.4±2.6
**c2**	-3.3±0.2	22.4±0.6	102.5±7.3
***Leuconostoc* species**	-3.4±0.2	24.2±1.0	96.3±7.8

S6 Table. R^2 of the calibration curves

**Assay**	**Calibration curve R^2**
936	0.99±0.01
P335	0.99±0.01
c2	0.99±0.01
Leuconostoc species	0.99±0.00

S7 Table. The linear dynamic range of the qPCR assays

**Assay**	**Linear dynamic range per reaction***
936	10^5 - 10^1
P335	10^5 - 10^1
c2	10^5 - 10^1
Leuconostoc species	10^5 - 10^1

*The linear dynamic range is expressed with reference to the number of DNA copies added to the PreAmp reactions, which consists of 3.75 µL of PreAmp master mix (Fluidigm, Product # 100-5580) plus 1.25 µL of pooled standard DNA mix (PSM).

Evidence for limit of detection (LOD)

The LOD of the system corresponds to approximately 10^1 DNA copies per PreAmp reaction. When the sample was diluted further:

- The amplification plots would assume considerably higher Ct values than expected
- The data quality decreases
- The standard deviation of technical and biological replicates increases
- More than five percent of the reactions are failed from being detected

9. Data analysis

qPCR analysis program and data treatment

Assays were run on the Biomark HD system (Fluidigm) and data was collected with the Biomark data collection Software. Data was eventually analyzed on Fluidigm Real-Time PCR Analysis Software v 4.1.3 [settings: quality threshold of 0.5, linear (derivative) baseline correction, and user-defined (global) Ct threshold method with EvaGreen threshold of 0.04]. The Modified Thompson Tau test was used to detect and dispose outliers.

Results for NTCs

S8 Table. Ct values, qualities and EvaGreen call for NTCs. Each three consecutive samples represent technical replicates of a given NTC.

Assay	Ct value	Quality	Call
sk1	23.3	1.0	Pass
sk1	22.9	1.0	Pass
sk1	21.6	1.0	Pass
sk1	26.7	0.8	Pass
sk1	27.8	0.8	Pass
sk1	999.0	0.0	Fail
sk1	999.0	0.0	Fail

sk1	999.0	0.0	Fail
sk1	999.0	0.0	Fail
sk1	999.0	0.0	Fail
sk1	999.0	0.0	Fail
sk1	999.0	0.0	Fail
sk1	999.0	0.0	Fail
sk1	999.0	0.0	Fail
sk1	999.0	0.0	Fail
sk1	999.0	0.0	Fail
sk1	999.0	0.0	Fail
sk1	999.0	0.0	Fail
sk1	999.0	0.0	Fail
sk1	999.0	0.0	Fail
sk1	999.0	0.0	Fail
sk1	999.0	0.0	Fail
sk1	26.7	0.8	Pass
sk1	999.0	0.0	Fail
sk1	999.0	0.0	Fail
sk1	999.0	0.0	Fail
sk1	999.0	0.0	Fail
sk1	22.4	0.9	Pass
sk1	22.7	1.0	Pass
sk1	22.1	0.9	Pass
sk1	21.8	1.0	Pass
sk1	22.4	1.0	Pass
sk1	21.3	1.0	Pass
sk1	23.6	1.0	Pass
sk1	23.0	0.9	Pass
sk1	25.3	1.0	Pass
sk1	25.3	1.0	Pass
sk1	24.5	1.0	Pass
sk1	26.5	1.0	Pass
sk1	25.8	1.0	Pass

sk1	24.8	1.0	Pass
sk1	999.0	0.0	Fail
TP901-1	21.7	1.0	Pass
TP901-1	22.2	1.0	Pass
TP901-1	21.8	1.0	Pass
TP901-1	27.2	0.7	Pass
TP901-1	999.0	0.0	Fail
TP901-1	27.9	0.7	Pass
TP901-1	999.0	0.0	Fail
TP901-1	999.0	0.0	Fail
TP901-1	26.6	0.7	Pass
TP901-1	999.0	0.0	Fail
TP901-1	999.0	0.0	Fail
TP901-1	999.0	0.0	Fail
TP901-1	999.0	0.0	Fail
TP901-1	999.0	0.0	Fail
TP901-1	999.0	0.0	Fail
TP901-1	999.0	0.0	Fail
TP901-1	999.0	0.0	Fail
TP901-1	999.0	0.0	Fail
TP901-1	999.0	0.0	Fail
TP901-1	999.0	0.0	Fail
TP901-1	999.0	0.0	Fail
TP901-1	999.0	0.0	Fail
TP901-1	999.0	0.0	Fail
TP901-1	999.0	0.0	Fail
TP901-1	999.0	0.0	Fail
TP901-1	999.0	0.0	Fail
TP901-1	999.0	0.0	Fail
TP901-1	33.4	0.9	Pass
TP901-1	24.0	0.9	Pass
TP901-1	999.0	0.0	Fail
TP901-1	23.5	1.0	Pass

TP901-1	24.6	1.0	Pass
TP901-1	22.1	0.9	Pass
TP901-1	26.7	1.0	Pass
TP901-1	999.0	0.0	Fail
TP901-1	999.0	0.0	Fail
TP901-1	25.9	1.0	Pass
TP901-1	25.8	1.0	Pass
TP901-1	999.0	0.0	Fail
TP901-1	23.1	1.0	Pass
TP901-1	21.6	1.0	Pass
TP901-1	23.0	1.0	Pass
P220	21.5	1.0	Pass
P220	23.9	1.0	Pass
P220	23.3	1.0	Pass
P220	999.0	0.0	Fail
P220	999.0	0.0	Fail
P220	999.0	0.0	Fail
P220	999.0	0.0	Fail
P220	999.0	0.0	Fail
P220	999.0	0.0	Fail
P220	999.0	0.0	Fail
P220	999.0	0.0	Fail
P220	999.0	0.0	Fail
P220	999.0	0.0	Fail
P220	999.0	0.0	Fail
P220	999.0	0.0	Fail
P220	999.0	0.0	Fail
P220	999.0	0.0	Fail
P220	999.0	0.0	Fail
P220	999.0	0.0	Fail
P220	999.0	0.0	Fail
P220	35.5	0.9	Pass
P220	999.0	0.0	Fail

P220	999.0	0.0	Fail
P220	999.0	0.0	Fail
P220	999.0	0.0	Fail
P220	999.0	0.0	Fail
P220	999.0	0.0	Fail
P220	23.8	1.0	Pass
P220	22.8	1.0	Pass
P220	22.6	1.0	Pass
P220	999.0	0.0	Fail
P220	999.0	0.0	Fail
P220	999.0	0.0	Fail
P220	999.0	0.0	Fail
P220	999.0	0.0	Fail
P220	999.0	0.0	Fail
P220	999.0	0.0	Fail
P220	999.0	0.0	Fail
P220	999.0	0.0	Fail
P220	999.0	0.0	Fail
P220	999.0	0.0	Fail
P220	999.0	0.0	Fail
LN04	999.0	0.0	Fail
LN04	25.0	1.0	Pass
LN04	999.0	0.0	Fail
LN04	999.0	0.0	Fail
LN04	999.0	0.0	Fail
LN04	999.0	0.0	Fail
LN04	999.0	0.0	Fail
LN04	999.0	0.0	Fail
LN04	999.0	0.0	Fail
LN04	999.0	0.0	Fail
LN04	999.0	0.0	Fail
LN04	999.0	0.0	Fail
LN04	999.0	0.0	Fail

LN04	999.0	0.0	Fail
LN04	999.0	0.0	Fail
LN04	999.0	0.0	Fail
LN04	999.0	0.0	Fail
LN04	999.0	0.0	Fail
LN04	999.0	0.0	Fail
LN04	999.0	0.0	Fail
LN04	999.0	0.0	Fail
LN04	999.0	0.0	Fail
LN04	999.0	0.0	Fail
LN04	999.0	0.0	Fail
LN04	999.0	0.0	Fail
LN04	999.0	0.0	Fail
LN04	999.0	0.0	Fail
LN04	23.2	1.0	Pass
LN04	24.8	1.0	Pass
LN04	23.2	1.0	Pass
LN04	24.2	1.0	Pass
LN04	23.3	1.0	Pass
LN04	23.8	1.0	Pass
LN04	999.0	0.0	Fail
LN04	999.0	0.0	Fail
LN04	999.0	0.0	Fail
LN04	999.0	0.0	Fail
LN04	999.0	0.0	Fail
LN04	999.0	0.0	Fail
LN04	999.0	0.0	Fail
LN04	999.0	0.0	Fail
LN04	999.0	0.0	Fail

Note: In the BioMark qPCR system, samples with no signals across the given number of cycles (40 in our case) are, by default, assigned a Ct value of 999.

Description of normalization method

The assays did not involve normalization with reference genes. However, as has been described, calibration curves constructed from serial ten-fold dilution of equimolar concentrations of DNA from known phages (sk1, TP90-1, P220 and phiLN04 representing 936, P335, c2 and *Leuconostoc* species of phages, respectively) were constructed and analyzed.

Number and stage of technical replicates

All the qPCR assays were analyzed as technical triplicates prepared from pre-amplified and exonuclease I (New England Biolabs, Cat #M0293L) treated samples.

Repeatability (intraassay variation)

<u>*qPCR assays for 936 phages*</u>

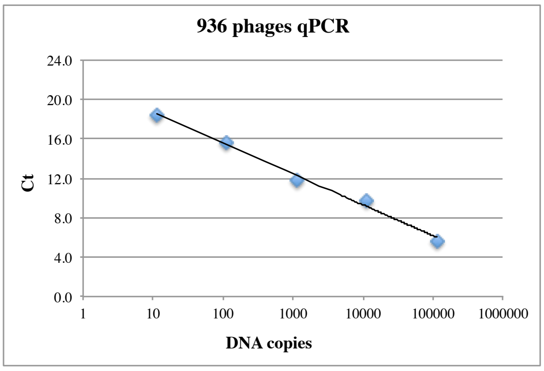

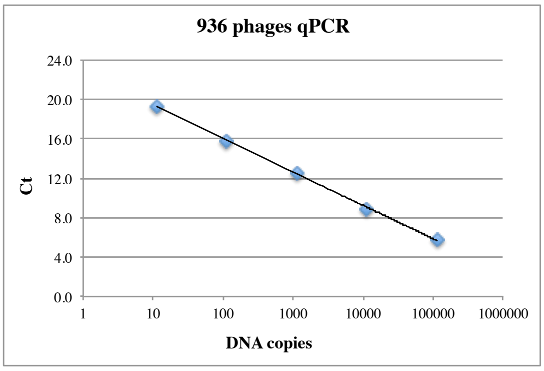

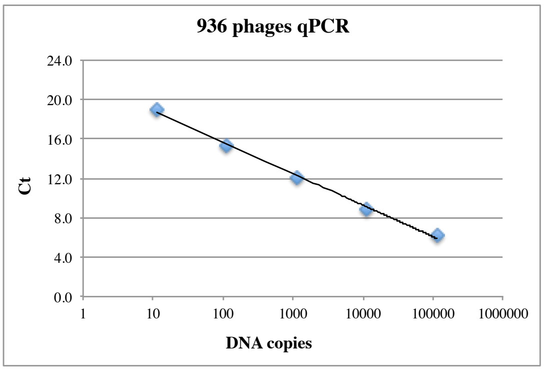

qPCR assays for P335 phages

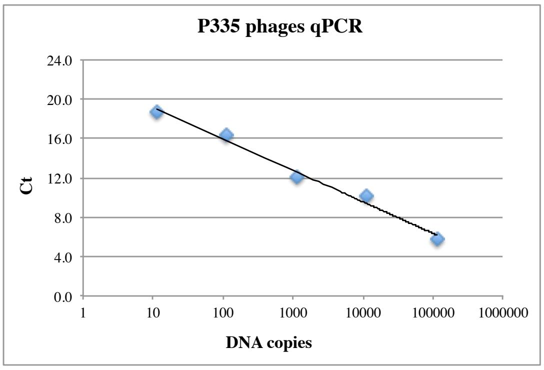

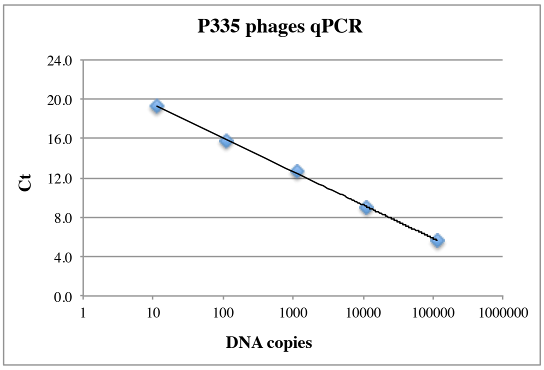

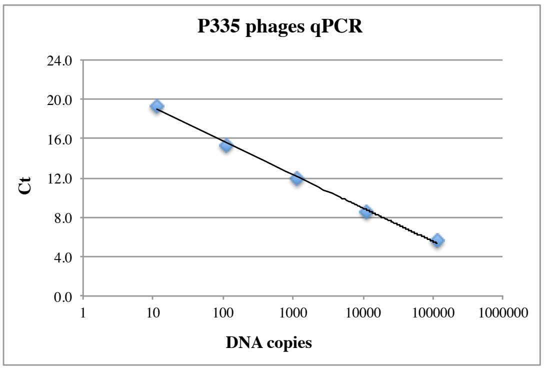

qPCR assays for c2 phages

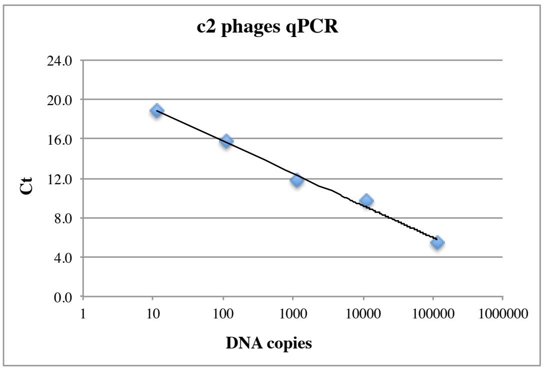

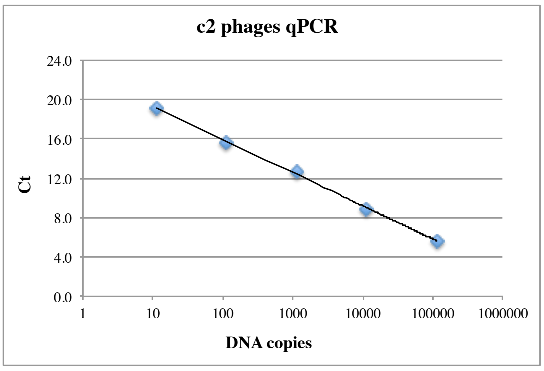

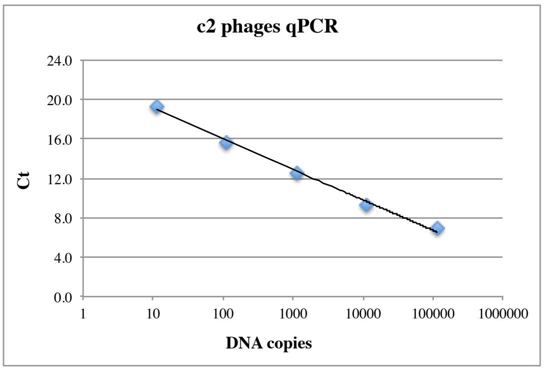

qPCR assays for Leuconostoc species phages

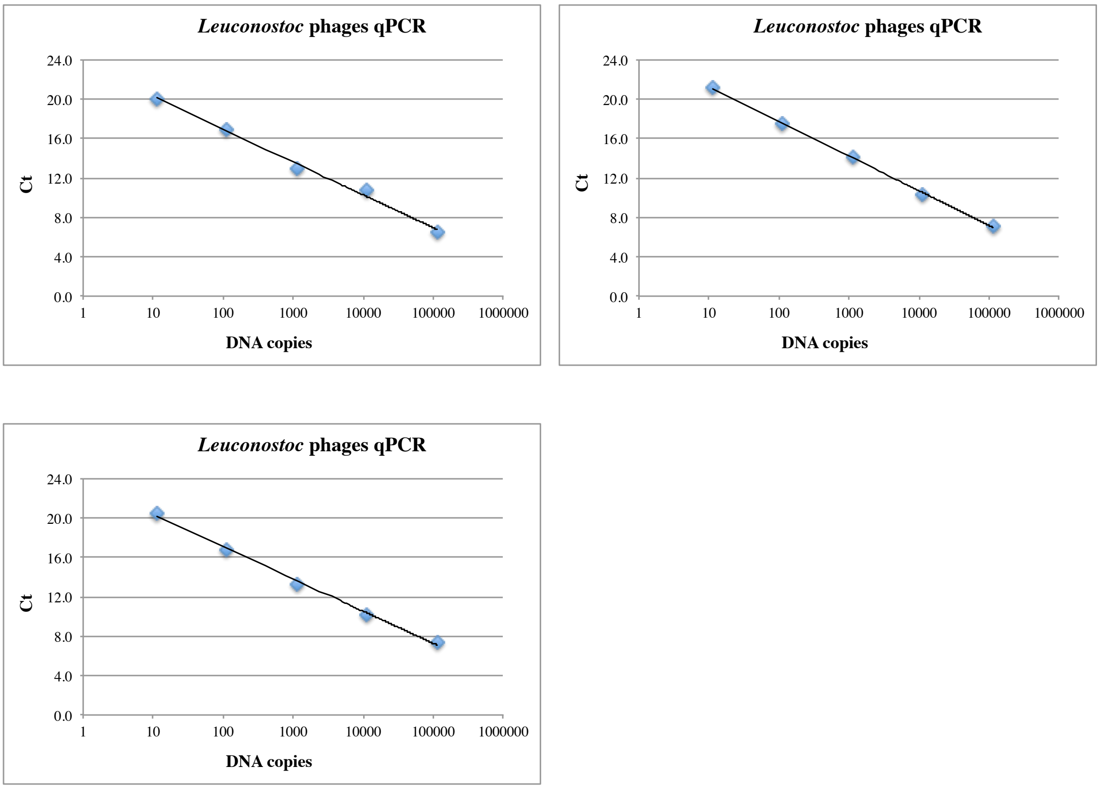

S5 Fig. Calibration curves showing the variations in Ct values within a given qPCR assay (intraassay variation). Variation is plotted as standard deviations of the Ct values of technical triplicates but it can be hardly seen because of the very small variation. Three plots per each phage assay correspond to the three independent experiments.

Reproducibility

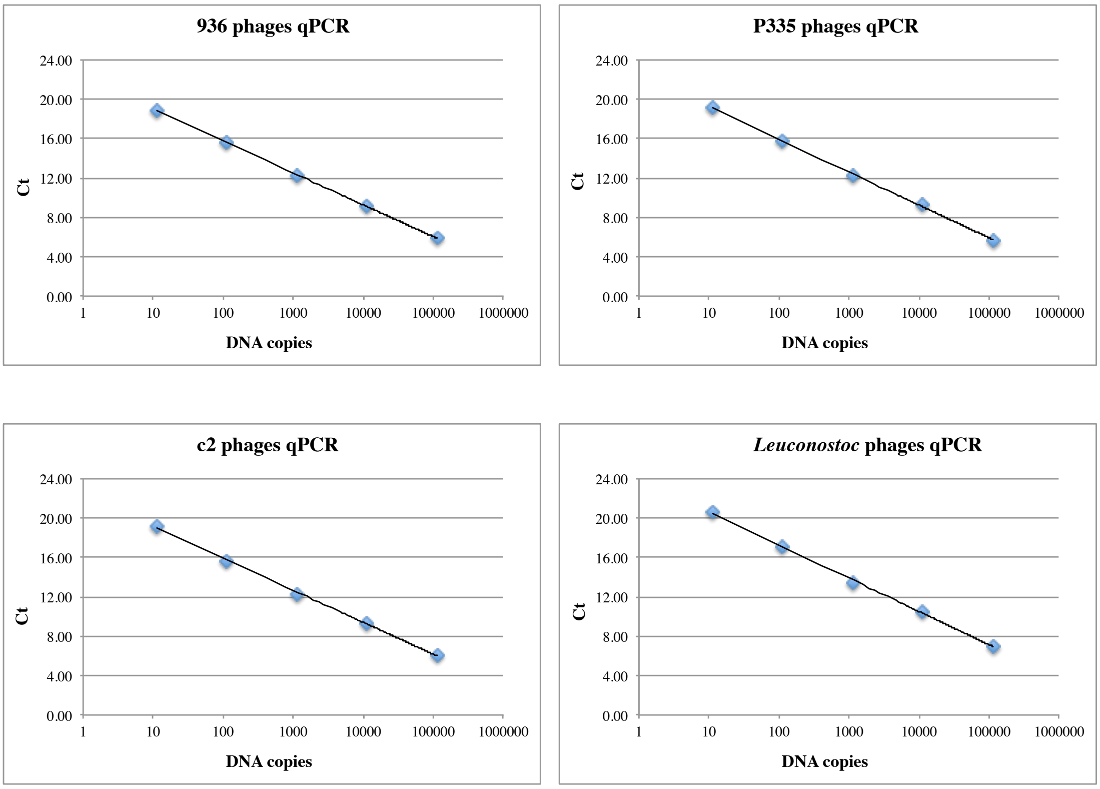

S6 Fig. Calibration curves constructed from plotting of the Ct values of the amplification plots against the estimated initial amounts of DNA molecules added per reaction. Although the variation is plotted as the standard deviations of the Ct values of the three independent experiments, it is hard to see because of the very small variation.

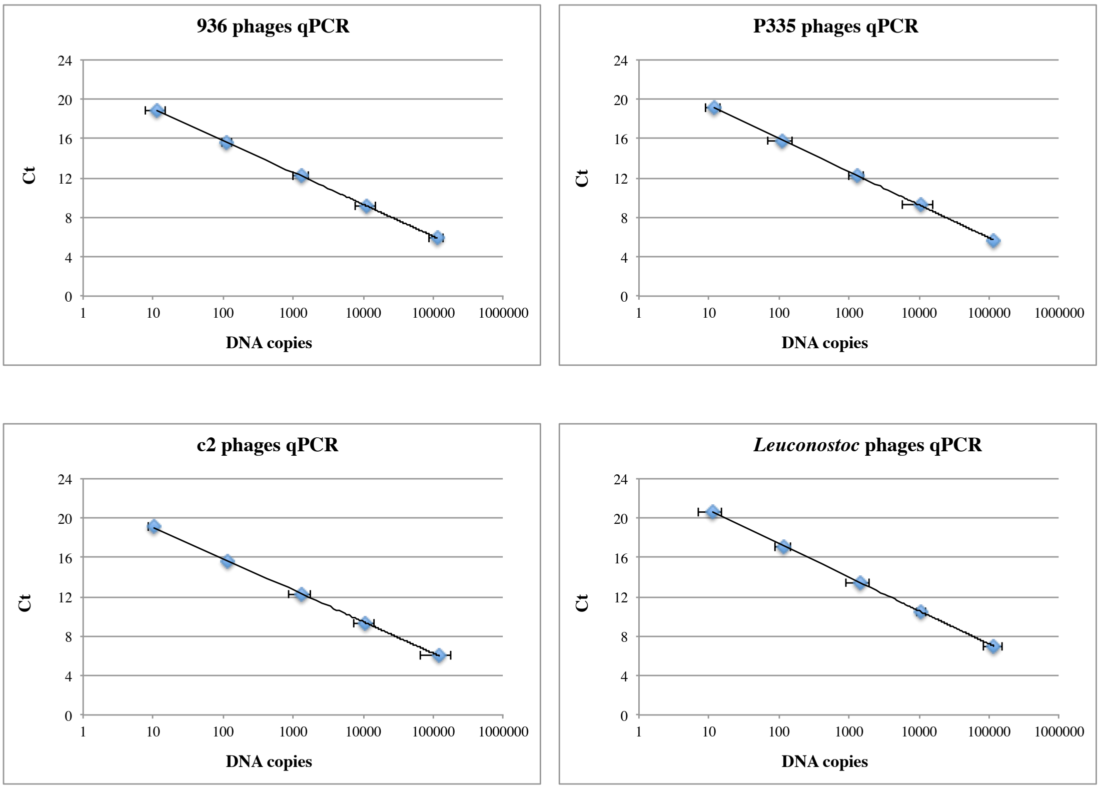

S7 Fig. Reproducibility (interassay variation). Calibration curves were constructed from plotting of the average number of DNA copies from three independent experiments against the corresponding average Ct values. Variation is expressed as the standard deviations of the average numbers of DNA copies detected. The target phage group that was being analyzed is indicated on top of each chart.

Statistical methods for results significance

One-way ANOVA

Softwares

StatPlus:mac LE Build 6.0.3/Core v5.9.92 and Microsoft® Excel® for Mac 2011 version 14.6.7
